# Mapping maternal mortality rate via spatial zero-inflated models for count data: A case study of facility-based maternal deaths from Mozambique

**DOI:** 10.1371/journal.pone.0202186

**Published:** 2018-11-09

**Authors:** Osvaldo Loquiha, Niel Hens, Leonardo Chavane, Marleen Temmerman, Nafissa Osman, Christel Faes, Marc Aerts

**Affiliations:** 1 Department of Mathematics and Informatics, Faculty of Sciences, Universidade Eduardo Mondlane, Maputo, Mozambique; 2 I-BioStat, Hasselt University, Diepenbeek, Belgium; 3 Centre for Health Economic Research and Modelling Infectious Diseases, Vaccine and Infectious Disease Institute (VAXINFECTIO), University of Antwerp, Antwerp, Belgium; 4 Epidemiology and Social Medicine (ESOC), Faculty of Medicine and Health Sciences, University of Antwerp, Antwerp, Belgium; 5 Jhpiego, Maputo, Mozambique; 6 International Centre for Reproductive Health, Ghent University, Ghent, Belgium; 7 Centre of Excellence Women and Child Health, Aga Kan University, Nairobi, Kenya; 8 Department of Obstetrics and Gynaecology, Maputo Central Hospital, Maputo, Mozambique; 9 Faculty of Medicine, Eduardo Mondlane University, Maputo, Mozambique; Johns Hopkins Bloomberg School of Public Health, UNITED STATES

## Abstract

Maternal mortality remains very high in Mozambique, with estimates from 2015 showing a maternal mortality ratio of 489 deaths per 100,000 live births, even though the rates tend to decrease since 1990. Pregnancy related hemorrhage, gestational hypertension and diseases such as malaria and HIV/AIDS are amongst the leading causes of maternal death in Mozambique, and a significant number of these deaths occur within health facilities. Often, the analysis of data on maternal mortality involves the use of counts of maternal deaths as outcome variable. Previously we showed that a class of hierarchical zero-inflated models were very successful in dealing with overdispersion and clustered counts when analyzing data on maternal deaths and related risk factors within health facilities in Mozambique. This paper aims at providing additional insights over previous analyses and presents an extension of such models to account for spatial variation in a disease mapping framework of facility-based maternal mortality in Mozambique.

## Introduction

Maternal mortality is still a major health problem in Mozambique, despite the country had registered significant advancements in the last 10 years with an annual reduction of approximately 4.4%, between 2005 and 2015 [1]. Although both direct (hemorrhage, eclampsia, puerperal infection, etc) and indirect (malaria, anemia, tuberculosis, HIV/AIDS, etc) complications have been pinpointed as the main causes of maternal deaths in the country [2–5], one important determinant continues to be the lack of infrastructure and human resources, as shown by the number of avoidable deaths within health facilities if appropriate care were provided [6].

Consider, for instance, the data in the Needs for Maternal and Neonatal Health (NMNH) survey [7] which motivates this study, where information was gathered from a random sample of 450 health facilities (HFs) from 126 randomly selected districts in 11 provinces of Mozambique. There were 278,173 obstetric admissions which resulted in 1,857 recorded maternal deaths. About 68% of deaths were due to direct obstetric complications and 32% caused by non-obstetric complications. The coverage of institutional deliveries is estimated at 58% [8] while the number of confirmed maternal deaths is 8 times higher than that reported by health facilities [9]. In addition, there is a considerable difference in access and quality of care services between rural and urban areas. Most rural health centers do not have qualified medical personnel and equipment for basic or comprehensive emergency obstetric care or lack established routines for assessment of the quality of maternity care offered [9, 10], which in many situations requires referrals of patients to “better” or urban health facilities.

**Fig 1:**
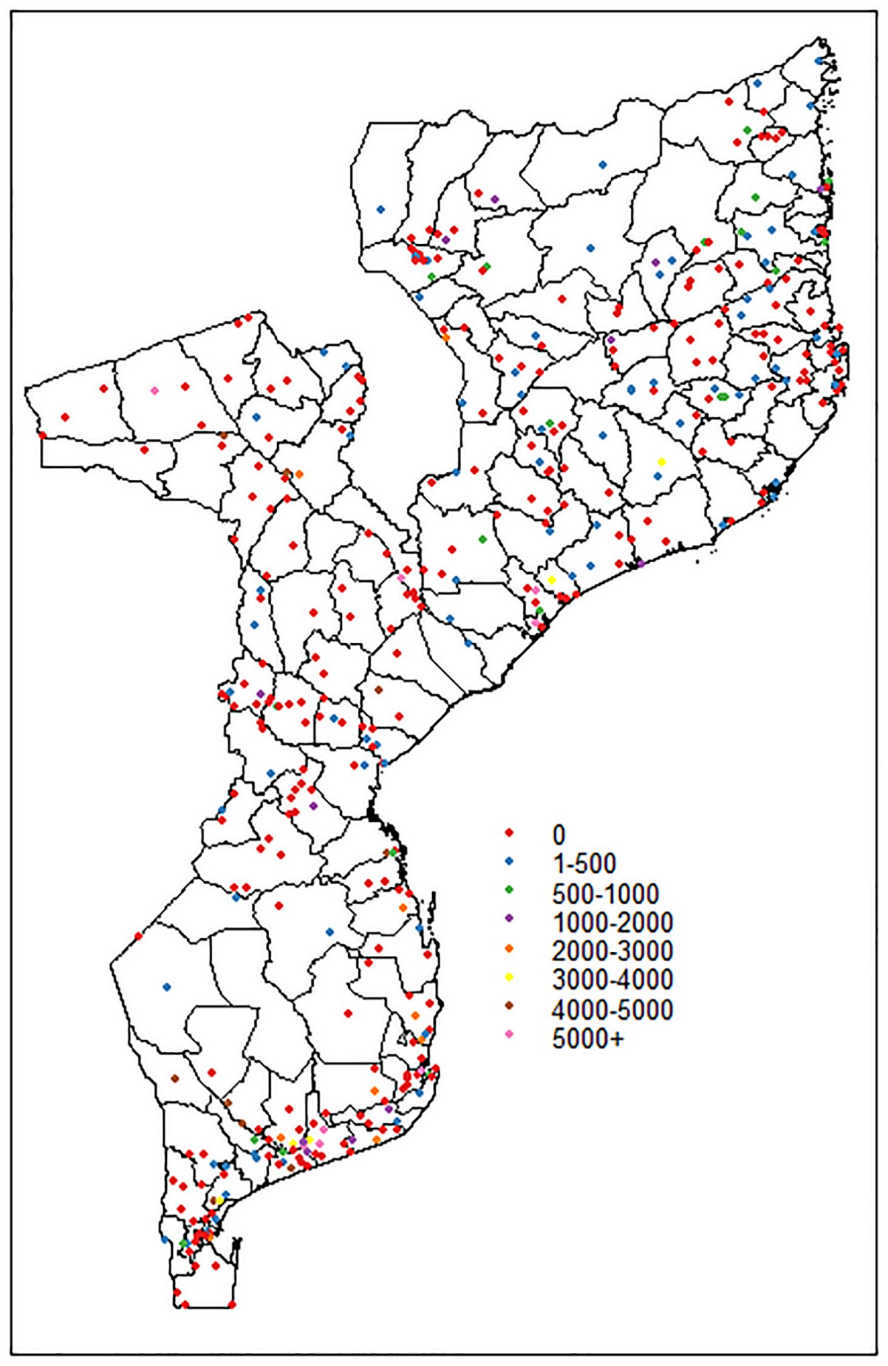
Map of facility-based maternal mortality rates per 100,000 obstetric admissions in Mozambique (2006-2007), based on the NMNH data.

For instance, in the NMNH survey, only 7.7% of maternal deaths were registered in health centers of class 2 (health centers type II, III and health posts), representing about 64% of all HFs sampled, which also included class 1 centers (hospitals and health center type I), much larger and located in the cities or district capital. Class 2 HFs were responsible for approximately 87.5% of referrals due to obstetric complications to class 1 HFs. The referrals from one facility to another may imply that no maternal deaths are reported in vast areas of the country. Fig 1 shows the map of facility-based maternal mortality ratio, per 100,000 obstetric admissions, leading to a phenomenon which appears quite often in count data collected in health services: the excessive number of zero counts, more than expected relative to the commonly used Poisson distribution.

**Fig 2:**
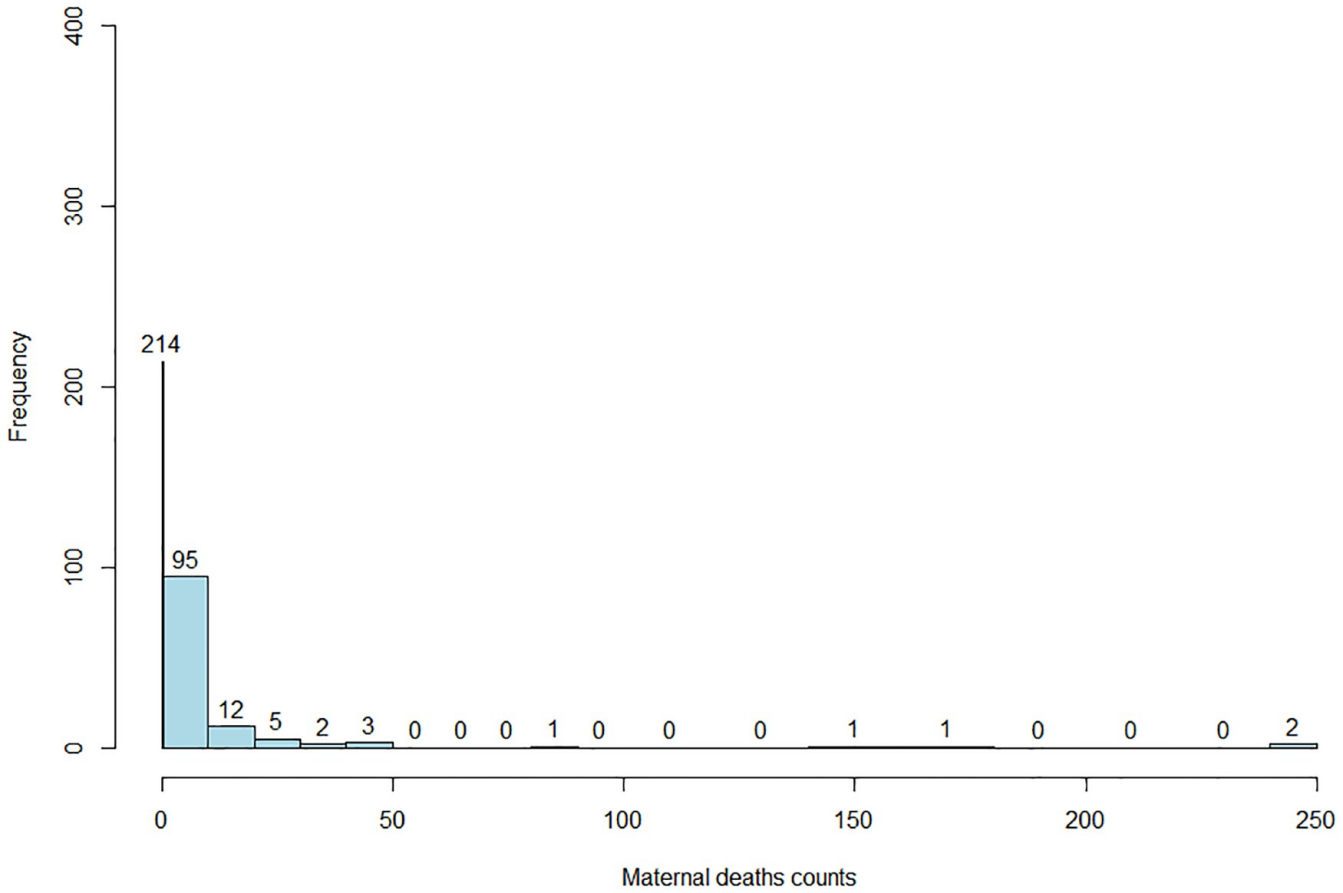
Histogram of observed facility-based maternal deaths in Mozambique (2006-2007), based on the NMNH data.

The histogram of observed maternal deaths in Fig 2 shows that about 63% of the 336 HFs reported zero maternal deaths.

Zero-inflated Poisson (ZIP) or Zero-inflated Negative Binomial (ZINB) and Hurdle models have been proposed to model data with extra zeros. They both assume that for each observation there are two possible data generating processes with different probabilities: one generates the zeros with probability *p* and another the counts with probability (1 − *p*). A Bernoulli model is used to determine which of the two processes is used. While the zero-inflated model assumes two types of zeros exists in the data (structural zeros and sampling zeros), the Hurdle model is a two-part conditional model which assumes that all zero data are from one “structural” source and the non-zero data have “sampling” origin following either a truncated Poisson or truncated negative binomial distribution [11, 12]. Since in the NMNH survey data, one should expect true zero maternal deaths to be reported in health centers lacking any surgery facility or maternity ward such as health centers of type III and health posts, and sampling zeros from health facilities of class 1 (provincial or district hospitals), zero-inflated models should be preferred to Hurdle models, which are more appropriate only when a true separation in the data generation process is known. There are many examples of applications of zero-inflated models in public health and social sciences [13–15], in ecological studies [16, 17] and other disciplines [18–20].

For lattice spatial count data, defined as spatially-indexed data associated with geographic regions or areas and a random variable for each area, hierarchical Poisson models are often used and easily implemented using the Bayesian framework [21, 22]. ZIP models have been extensively applied in the Bayesian context [23–25], as well as its spatial counterpart with applications in ecological [26] and health fields [27–30].

Usually, spatial heterogeneity is accounted for by introducing Gaussian random effects such as the Conditional Autoregressive model (CAR) either in the non-zero component of the model or in both model components via a bivariate CAR model. The former case is well illustrated by Agarwal *et al*. [31] who applied a ZIP model to counts of isopod nest burrows in Israel and by Gschlöb and Czado [32] who present a review of models for count data with overdispersion and spatial effects applied to the number of invasive meningococcal disease cases in Germany. On the other hand, Neelon *et al*. [25] used a Hurdle model with bivariate CAR prior for spatial random effects introduced on both model components (i.e., dependence between components), and applied to health services data. Although less commonly encountered in the spatial zero-inflated literature, allowing for between-component correlation reduces bias in parameter estimates, and can be easily fitted in the Bayesian context using standard software [25].

A result from Loquiha *et al*. [33], based on a ZINB model with shared random effects, showed that in the North of Mozambique, HFs located outside the district capital had a lower estimated value for mortality rate; the same holds for HFs in the Center but less pronounced, and for HFs in the South there was no difference between HFs in the district capital or outside. To test whether facility-based mortality rate was spatially different across areas in Mozambique, we considered extending the zero-inflated models previously used for these data, expecting to observe clusters of areas with elevated or reduced mortality rate between the North, Center and South of Mozambique. Our approach considers the inclusion of spatially indexed random effects to accommodate unmeasured within and between-component spatial dependence on a set of hierarchical ZIP models (non-spatial normal random effects), in a Bayesian context. This enables the models to deal with both non-spatial and spatial clusters due to common environmental, demographic or cultural effects shared by neighboring areas, improving our understanding of spatial patterns and differences in mortality rates across areas [34]. We will refer to these models as spatial ZIP or spatial ZINB.

Due to the complexity of the posterior distribution for parameter estimation, we relied on MCMC algorithms implemented in the WinBUGS software (version 14.0), which contrary to the recent Integrated Nested Laplace Approximation (INLA) method, allows fitting a regression model for the zero-inflation component [29]. Model comparison was done using DIC and Brier score as suggested in Gschlöb and Czado [32].

The remaining of this paper is organized as follows: details of the NMNH survey are provided in the next section with some descriptive statistics of the variables used in this study, followed by an introduction of the zero-inflated model and its extensions to account for non-spatial and spatial heterogeneity. Model estimation and selection are discussed in the fourth section. The fifth section presents the results of the application of the models to the NMNH survey data. The paper ends with a discussion of the results.

## The NMNH survey

The Needs for Maternal and Neonatal Health (NMNH) survey is a nationwide survey at the level of HFs, conducted from November 1/2006 to October 31/2007 by the Mozambican Ministry of Health, in order to provide the health authorities with an assessment of the progress in controlling and decreasing maternal and neonatal mortality within the HFs as well as with an assessment of the availability of infrastructures and other resources for the management of maternal obstetric and newborn complications [7]. The NMNH survey data file is available from S1 File.

Besides the number of maternal deaths, the following information was available at health facility level: region (North, Center and South), location of HF (inside or outside district capital), type of HF (central hospital, general hospital, health centers I, II,III and health posts), existence of emergency obstetric care (yes or no), waiting house (or room, yes or no), proportion of HIV and malaria cases (among obstetric admissions), ratio of medical doctors (among the medical staff) and proportion of referrals from and to the HF. Due to missing data, out of the 450 HF and 126 districts records were complete for 336 HF from 124 districts, excluding the districts of Chigubo and Chinde (not included in the survey), and Tambara and Malema (missing data), with a maximum of 10 HFs in a given district and nearly 63% of HFs reported 0 maternal deaths. The average number of maternal deaths equaled 5.33, with variance 510.25 (see Table 1). The proportion of HIV and malaria cases in the HFs was on average 0.0120 and 0.0125, respectively. The average ratio of medical doctors was equal to 0.473, with majority of HFs of Type II/III/health post (64.1%), next to central hospitals (0.6%), provincial and general hospitals (2.7%), Type I HFs (27%), and rural hospitals (5.6%). Obstetric emergency care was full time available in 53.6% of the HFs, and a waiting house was available in only 27.6%. The geographical distribution of the HFs was as follows: 34.7% in the North, 32.6% in the Center and 32.6% in the South; while only 35% of the HFs were located inside the district capitals.

**Table 1:** Summary statistics of facility-based maternal deaths and rates per 100,000 obstetric admissions in Mozambique (2006–2007).

Statistcs	Maternal deaths	Mortality rate
*mean*	5.33	504.67
*standard deviation*	22.59	840.33
*median*	0	207.37
*mininum*	0	0
*maximum*	244	4752.85

**Fig 3:**
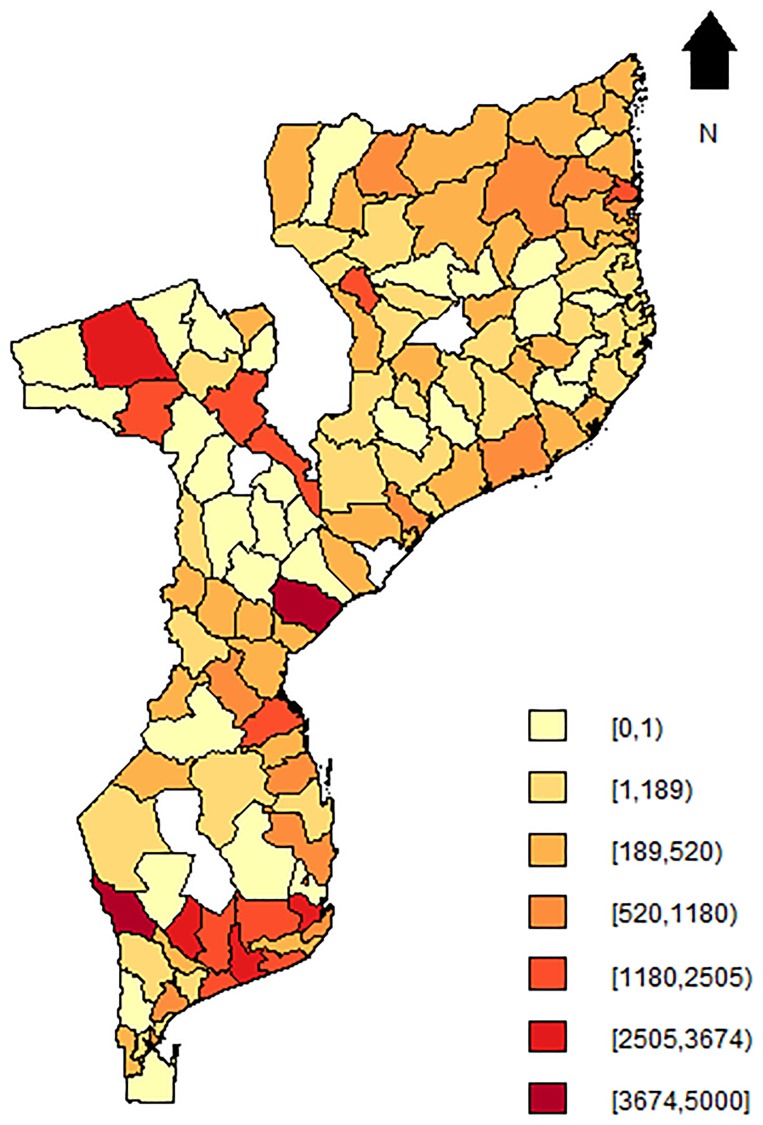
Map of facility-based maternal mortality rates per 100,000 obstetric admissions in Mozambique (2006-2007) at district-level. Blank spots indicate districts for which data was not available.

Fig 3 shows the observed institutional maternal mortality rate at the district level, obtained after aggregating the observed counts and dividing by the total number of obstetric admissions within each district (multiplied by 100,000), with obstetric admissions used as a proxy for the total number of women at risk of maternal death. The mean mortality rate was 504.67 (per 100,000 obstetric admissions), standard deviation of 840 and median of 207.37 (range: 0.0—4752.85). Geographically, the highest rates were found in the South, where districts of Gaza and Inhambane, located alongside the coastal line of Mozambique such as Chibuto, Manjacazi or Homoine, had rates greater than 3000 (per 100,000 obstetric admissions). The district of Muanza in the province of Sofala province had a rate larger than 4000 (per 100,000 obstetric admissions) and the districts of Maravia, Moatize and Cahora-Bassa in the province of Tete, had rates larger than 2500 (per 100,000 obstetric admissions), constituting the highest cases in central Mozambique. In the North, the highest rates were mostly observed in the Cabo Delgado province and were not more than 2500 (per 100,000 obstetric admissions). The highest institutional maternal mortality rate was observed in the district of Massingir in the southwest of the Gaza province, with 4752.9 (per 100,000 obstetric admissions), i.e, 25 maternal deaths among 526 obstetric admissions, for a district with a population density of 4.8 persons per km^2^ according to the 2007 population census [35]. In the next section, we describe the different statistical models that will be applied to the NMNH data.

## Zero-inflated models

### Hierarchical zero-inflated models

Let *y*_*ij*_, *N*_*ij*_ and ${\hat{\lambda }}_{ij}$ be the number of maternal deaths, obstetric admissions (population at risk) and observed mortality rate (${\hat{\lambda }}_{ij}=\frac{y_{ij}}{{\text{N}}_{ij}}$) for district *i* and health facility *j* (*i* = 1, …, *n*; *j* = 1, …, *n*_*i*_), and let ***x**_ij_* and ***z**_ij_* denote two sets of explanatory variables or risk factors. A zero-inflated (ZI) distribution is defined as follows

$$\begin{array}{c} y_{ij}∼\{\begin{array}{cc} f(y_{ij}) & \text{with}\;\text{probability}\;1-\pi_{ij}\text{,}\\ 0 & \text{with}\;\text{probability}\;\pi_{ij}\text{.} \end{array} \end{array}$$ (1)

Two ZI distributions are considered for this application: with a Poisson (P) or negative binomial (NB) distribution for *f*(*y*_*ij*_) and *π*_*ij*_ the zero-inflation probability. Denote by λ_*ij*_ the mortality rate and *ϕ* the dispersion parameter of the Negative Binomial distribution, then we can rewrite (1) as

$$\begin{array}{c} y_{ij}|{\text{N}}_{ij},\lambda_{ij},\pi_{ij}∼\text{ZIP}\left({\text{N}}_{ij}\lambda_{ij},\pi_{ij}\right), \end{array}$$

for the ZIP distribution, or

$$\begin{array}{c} y_{ij}|{\text{N}}_{ij},\lambda_{ij},\pi_{ij},\phi ∼\text{ZINB}\left({\text{N}}_{ij}\lambda_{ij},\pi_{ij},\phi \right), \end{array}$$

for the ZINB distribution.

Denoting *ν*_*ij*_ = *N*_*ij*_λ_*ij*_, the parameters *ν*_*ij*_ and *π*_*ij*_ can be modeled as a function of covariates ***x***_*ij*_ and ***z***_*ij*_ using canonical link functions:

$$\begin{array}{c} \text{logit}\left(\pi_{ij}\right)=z_{ij}^{t}\alpha ,\\ \text{log}\left(\nu_{ij}\right)=x_{ij}^{t}\beta +\text{log}\left({\text{N}}_{ij}\right), \end{array}$$ (2)

where ***α*** and ***β*** are vectors of model parameters of length *q*_*α*_ and *q*_*β*_ parameters, respectively. The mean of *y*_*ij*_ is given by

$$\begin{array}{c} \mu_{ij}=\text{E}\left(y_{ij}|x_{ij},z_{ij}\right)=\left(1-\pi_{ij}\right)\nu_{ij}. \end{array}$$

If data is hierarchically structured, such as in the NMNH survey with health centers clustered within districts, Hall [36] introduced the ZIP model with random effects, which we will refer to by adding H (Hierarchical) to the ZIP and ZINB acronym, i.e., HZIP and HZINB, respectively. Model (2) now turns into

$$\begin{array}{c} \begin{array}{c} \text{logit}\left(\pi_{ij}\right)=z_{ij}^{t}\alpha +\vartheta_{i},\\ \text{log}\left(\nu_{ij}\right)=x_{ij}^{t}\beta +\text{log}\left({\text{N}}_{ij}\right)+\theta_{i}, \end{array} \end{array}$$ (3)

where *θ*_*i*_ and *ϑ*_*i*_ are random intercepts for the *i*-th district usually assumed to be

$$\begin{array}{c} (\begin{array}{c} \theta_{i}\\ \vartheta_{i} \end{array})∼N_{2}(0,\Sigma_{\theta }),\Sigma_{\theta }=(\begin{array}{cc} \sigma_{\theta }^{2} & \rho \sigma_{\theta }\sigma_{\vartheta }\\ \rho \sigma_{\theta }\sigma_{\vartheta } & \sigma_{\vartheta }^{2} \end{array}), \end{array}$$

with *ρ* the between-components correlation parameter, i.e., the correlation between the zero-inflation probability (on the logit scale) and the mean number of deaths (on the log scale) across the districts. Higher values for *ϑ*_*i*_ are indicative of a higher probability of zero maternal deaths in district *i* compared to other districts. Similarly, higher values for *θ*_*i*_ imply larger expected counts of maternal deaths in district *i* compared to other districts. With this model specification, district effects on the maternal mortality rate can be accounted for via the random effects *θ*_*i*_ and *ϑ*_*i*_. It also allows a multitude of parameterizations for the covariance matrix structure, such as the shared parameter model if we let *ϑ_i_* = *ςθ_i_*, for some proportionality constant *ς*, implying that $\sigma_{\vartheta }^{2}=\varsigma^{2}\sigma_{\theta }^{2}$, or the independent random intercepts model when *ρ* = 0. The case where *ρ*^2^ ≠ 0 and *ρ*^2^ ≠ 1 will be referred to as HZIP or HZINB (correlated) and for *ρ* = 0 and *ϑ_i_* = *ςθ_i_* as HZIP (independence) and HZIP (shared), respectively. We showed previously in Loquiha *et al*. [33], using likelihood-based methods, that the HZINB (shared) provided better fit to the NMNH data and that the negative binomial family of models outperformed its Poisson counterpart.

### Spatial zero-inflated models

We now extend model (3) to accommodate both non-spatially and spatially structured heterogeneity. Let *θ*_*i*_ and *ϑ*_*i*_ be the non-spatially and *υ*_*i*_ the spatially structured random effects for the *i*-th district. The model can be written as

$$\begin{array}{c} \begin{array}{c} \text{logit}\left(\pi_{ij}\right)=z_{ij}^{t}\alpha +\vartheta_{i},\\ \text{log}\left(\nu_{ij}\right)=x_{ij}^{t}\beta +\text{log}\left({\text{N}}_{ij}\right)+\theta_{i}+\upsilon_{i} \end{array} \end{array}$$ (4)

For lattice data, spatial dependence between the counts is introduced via *υ*_*i*_, and usually one assumes the *υ*_*i*_ to follow a Conditional Autoregressive (CAR) model, a proper distribution defined as

$$\begin{array}{c} {\left(\upsilon_{i}|\upsilon_{i^{\prime }},\sigma_{\upsilon }^{2}\right)}_{i\neq i^{\prime }}∼N(\frac{\psi \sum_{i∼i^{\prime }}\upsilon_{i^{\prime }}\omega_{ii^{\prime }}}{\sum_{i∼i^{\prime }}\omega_{ii^{\prime }}},\frac{\sigma_{\upsilon }^{2}}{\sum_{i∼i^{\prime }}\omega_{ii^{\prime }}}), \end{array}$$ (5)

where *ω*_*ii*′_ = 1 if *i* and *i*′ are adjacent (or *i* ∼ *i*′) and 0 otherwise, and *ψ* is a spatial autocorrelation parameter.

If *ψ* = 1 in (5) then the intrinsic CAR model proposed by Besag *et al*. [37] is obtained. In WinBUGS version 14.0, intrinsic CAR can be specified via the car.normal function and proper CAR through the car.proper function. Similarly to the hierarchical situation in the previous section 1, the case where *ρ*^2^ ≠ 0 and *ρ*^2^ ≠ 1 will be referred as spatial hierarchical ZIP/ZINB (correlated), denoted SpHZIP/SpHZINB (correlated) and for *ρ* = 0 and *ϑ_i_* = *ςθ_i_* as SpHZIP (independence) and SpHZIP (shared) respectively.

Model (4) assumes that all correlation within and between-components is accounted for by the unstructured random intercepts *θ*_*i*_ and *ϑ*_*i*_ and thus the propensity for maternal deaths and number of maternal deaths are spatially unrelated. This is possibly not the case in the NMNH data, where clusters of areas more prone for maternal deaths are located in major cities along the coastal line (see Fig 1). To allow for this association due to unobserved common environmental or demographic effects and sharing of information across neighboring areas, a bivariate vector of spatially correlated data in each area or district, ***υ***_*i*_ = (*υ*_1*i*_, *υ*_2*i*_)^*t*^, *i* = 1, …, *n*, should be considered. We could extend model (4) towards

$$\begin{array}{c} \begin{array}{c} \text{logit}\left(\pi_{ij}\right)=z_{ij}^{t}\alpha +\vartheta_{i}+\upsilon_{1i},\\ \text{log}\left(\nu_{ij}\right)=x_{ij}^{t}\beta +\text{log}\left({\text{N}}_{ij}\right)+\theta_{i}+\upsilon_{2i}, \end{array} \end{array}$$ (6)

using an intrinsic bivariate CAR prior,

$$\begin{array}{c} \upsilon_{i}|\upsilon_{1(-i)},\upsilon_{2(-i)}∼N_{2}({\bar{\upsilon }}_{i},\frac{\Sigma_{\upsilon }}{\sum_{i∼i^{\prime }}\omega_{ii^{\prime }}}), \end{array}$$

where *υ*_1(−*i*)_, *υ*_2(−*i*)_ denotes the elements of ***υ*** excluding the *i*-th area, ${\bar{\upsilon }}_{i}=\left({\bar{\upsilon }}_{1i},{\bar{\upsilon }}_{2i}\right)$ and

$$\begin{array}{c} {\bar{\upsilon }}_{pi}=\frac{\sum_{i∼i^{\prime }}\upsilon_{pi^{\prime }}\omega_{ii^{\prime }}}{\sum_{i∼i^{\prime }}\omega_{ii^{\prime }}},\;\;\;p=1,2,\; \end{array}$$

while **Σ**_*υ*_ is a 2 × 2 covariance matrix with diagonal elements $\sigma_{\upsilon_{1}}^{2}$ and $\sigma_{\upsilon_{2}}^{2}$ representing the conditional variances of *υ*_1*i*_ and *υ*_2*i*_ respectively, and off-diagonal element $\sigma_{\upsilon_{12}}$ representing the conditional within-district covariance between *υ*_1*i*_ and *υ*_2*i*_, which controls the between-components spatial association. If $\sigma_{\upsilon_{12}}$ is positive then areas with a higher probability of maternal deaths will tend to show elevated numbers of facility-based maternal deaths, whilst $\sigma_{\upsilon_{12}}=0$ is indicative of spatially unrelated model components. The motivation of including the two random effects lies in the fact that the spatial dependence of the intrinsic CAR random effect is pre-determined by the neighborhood structure. Unstructured effects are included to allow for Bayesian learning about the strength of spatial dependence in the data, via the relative contributions of the two random effects to the posterior [37, 38]. Note also that (*θ*_*i*_, *ϑ*_*i*_) and ($\theta_{i^{{}^{\prime }}},\vartheta_{i^{{}^{\prime }}}$), as well as (*θ*_*i*_, *ϑ*_*i*_) and ($\upsilon_{1i^{{}^{\prime }}},\upsilon_{2i^{{}^{\prime }}}$) are assumed independent for any *i* ≠ *i*′.

We will denote this models spatial hierarchical ZIP (correlated-correlated) or SpHZIP (correlated-correlated) the case where *ρ* ≠ 0 and $\sigma_{\upsilon_{12}}\neq 0$, and as spatial hierarchical ZIP (correlated-independence) or SpHZIP (correlated-independence) if *ρ* ≠ 0 and $\sigma_{\upsilon_{12}}=0$. A good model building strategy suggests starting the fitting process with the SpHZIP (correlated-correlated) and if we fail to reject the hypothesis that $\sigma_{\upsilon_{12}}=0$, continue with the SpHZIP (correlated-independence) or with a further simplified version [25]. This can be easily implemented in standard Bayesian software, and although a proper multivariate CAR prior has been discussed elsewhere [39], only the intrinsic option is currently available in WinBUGS (or OpenBUGS), using the mv.car function.

## Model estimation and selection

Given the high dimensional and complex distributions for the models presented in the previous section, a Bayesian approach was considered for parameter estimation. The Bayesian context offers a flexible framework capable of accommodating complex relationships between data and models while incorporating various sources of uncertainty such as uncertainty about model parameters or missing data via prior distributions [21]. As such, we specified the negative binomial distribution as a Poisson-Gamma mixture model [40],

$$\begin{array}{c} y_{ij}|u_{ij}∼\text{Poisson}\left(\lambda_{ij}u_{ij}\right)\;\;\;\text{and}\;\;\;u_{ij}∼\text{Gamma}\left(r,r\right), \end{array}$$

where *y*_*ij*_ = 0, 1, 2, …, *r* and *r* > 0 is a positive parameter. Under this parametrization, the marginal distribution of *y* (discarding any subscript) is given by:

$$\begin{array}{c} f\left(y\right)=\int_{0}^{\infty }f\left(y|u\right)f\left(u\right)du=\frac{\Gamma (y+r)}{y!\Gamma (r)}{\left(\frac{r}{r+\lambda }\right)}^{r}{\left(\frac{\lambda }{r+\lambda }\right)}^{y}, \end{array}$$

which is a negative binomial distribution with parameters *r*/(*r* + λ) and *ϕ* = *r*^−1^.

Samples from the posterior distributions of model parameters were drawn using MCMC methods, specifically the Metropolis-Hastings algorithm. The following non-informative prior distributions were assigned to the model parameters:

$$\begin{array}{c} \begin{array}{c} \beta_{r}∼N\left(0,\sigma_{\beta_{r}}^{2}\right)\;\;\;\;\text{with}\;\;\;\;\sigma_{\beta_{r}}^{-2}∼\Gamma \left(10^{-5},10^{-5}\right)\;\;\;\text{and}\;\;\;r=1,2,\ldots ,q_{\beta },\\ \alpha_{t}∼N\left(0,\sigma_{\alpha_{t}}^{2}\right)\;\;\;\;\text{with}\;\;\;\;\sigma_{\alpha_{t}}^{-2}∼\Gamma \left(10^{-5},10^{-5}\right)\;\;\;\text{and}\;\;\;t=1,2,\ldots ,q_{\alpha },\\ \theta_{i}∼N\left(0,\sigma_{\theta }^{2}\right)\;\;\;\;\text{with}\;\;\;\;\sigma_{\theta }^{-2}∼\Gamma \left(10^{-3},10^{-3}\right),\\ \vartheta_{i}∼N\left(0,\sigma_{\vartheta }^{2}\right)\;\;\;\;\text{with}\;\;\;\;\sigma_{\vartheta }^{-2}∼\Gamma \left(10^{-3},10^{-3}\right),\\ \tau_{\upsilon }^{2}∼\Gamma \left(5\times 10^{-3},5\times 10^{-3}\right)\;\;\;\;\text{with}\;\;\;\sigma_{\upsilon }^{2}=\tau_{\upsilon }^{-2},\\ \tau_{\upsilon_{1}}^{2},\tau_{\upsilon_{2}}^{2}∼\Gamma \left(5\times 10^{-3},5\times 10^{-3}\right)\;\;\;\;\text{with}\;\;\;\sigma_{\upsilon_{1}}^{2}=\tau_{\upsilon_{1}}^{-2}\;\;\;\;\text{and}\;\;\;\;\sigma_{\upsilon_{2}}^{2}=\tau_{\upsilon_{2}}^{-2},\\ \phi =\frac{1}{r},\;\;\;r\;\text{being}\;\text{the}\;\text{order}\;\text{parameter}\;\text{in}\;\text{the}\;\text{NB}\;\text{distribution}\;\text{and}\;\;\;r∼\Gamma \left(10^{-3},10^{-3}\right),\\ \varsigma ∼N(0,10^{-4}). \end{array} \end{array}$$

A Wishart prior with 2 degrees of freedom was assumed for the inverse covariance matrix on the bivariate distribution for both the spatial and non-spatial random effects:

$$\begin{array}{c} \Sigma_{\theta }^{-1}∼\text{Wishart}\left(\Omega ,2\right)\;\;\;\text{and}\;\;\;\Sigma_{\upsilon }^{-1}∼\text{Wishart}\left(\Omega ,2\right), \end{array}$$

with **Ω** a scale matrix and a prior guess of the order of the covariance matrix,

$$\begin{array}{c} \Omega =(\begin{array}{cc} 1 & 0\\ 0 & 1 \end{array}). \end{array}$$

The “zero trick” strategy, which consists in using a well known distribution such as the Poisson distribution to indirectly specify an arbitrary model likelihood, was used to implement the ZIP and ZINB likelihood, since in WinBUGS no default likelihood currently exists for these distributions [40]. If we assume a model with log-likelihood *ℓ*_*ij*_ = log *f*(*y*_*ij*_|Θ), then using the “zero trick” strategy the model likelihood is written as

$$\begin{array}{c} f\left(y_{ij}|\Theta \right)=\prod_{i=1}^{n}\prod_{j=1}^{n_{i}}\exp\left(\ell_{ij}\right)=\prod_{i=1}^{n}\prod_{j=1}^{n_{i}}\frac{\exp\left(-\left(-\ell_{ij}\right)\right){\left(-\ell_{ij}\right)}^{0}}{0!}=\prod_{i=1}^{n}\prod_{j=1}^{n_{i}}f_{P}\left(0,-\ell_{ij}\right) \end{array}$$

where Θ is a set of parameters of interest and *f*_*P*_ the Poisson probability density function. To ensure the positivity of the likelihood, a positive constant *C* was added such that −*ℓ*_*ij*_ + *C* > 0. WinBUGS codes for this implementation are available in the S1 Appendix. A total of 50,000 iterations were used with a burn-in of 20,000 iterations. Convergence of MCMC chains was monitored using trace plots.

For selection of competing models we used DIC [41] which is given by

$$\begin{array}{c} DIC=2\bar{\text{D}(\Theta )}-\text{D}\left(\bar{\Theta }\right) \end{array}$$

where D denotes the Deviance and an over-line denotes the posterior expectation. One major weakness of DIC is that it lacks invariance to re-parameterizations due to the use of the posterior mean $\bar{\Theta }$, which should be chosen on computational grounds so to provide likelihoods that are available in closed forms [41, 42].

One alternative is to use a scoring measure such as the Brier score as discussed in Gschlößl and Czado [32], for categorical variables. The Brier score is a proper score such that the highest score is obtained for the best model. It is based on the posterior predictive probabilities

$$\begin{array}{c} p_{ij_{s}}=\text{P}\left(y_{ij}=s|\Theta \right). \end{array}$$

We used the following definition for the Brier score:

$$\begin{array}{c} -\frac{1}{n}\sum_{k=1}^{J}\sum_{i=1}^{n}\sum_{j=1}^{n_{i}}{\left(p_{ij_{s}}-{\hat{p}}_{ij_{s}}^{emp}\right)}^{2}, \end{array}$$

for *k* = 1, …, *J*, the k-th iteration of the MCMC algorithm and ${\hat{p}}_{ij_{s}}^{emp}=1$ if *y*_*ij*_ = *s* and 0 otherwise, the empirical probability that observation *ij* takes the value *s*. The higher the score, the better the model. To obtain the posterior predictive probabilities $p_{ij_{s}}=\text{P}\left(y_{ij}=s|\Theta \right)$, we used the posterior predictive ordinate or PPO [40],

$$\begin{array}{c} PPO_{ij}=P\left(y_{ij}=s|Y\right), \end{array}$$

estimated by

$$\begin{array}{c} {\hat{PPO}}_{ij}=\frac{1}{J}\sum_{k=1}^{J}P\left(y_{ij}=s|\Theta^{k}\right), \end{array}$$

with Θ^*k*^ the vector of parameter values generated in the *k*-th MCMC iteration. To calculate the $\hat{PPO}$ using the MCMC outputs one only needs to set a node equal to the likelihood evaluated at the current values of Θ.

## Application to the NMNH survey

The models considered for this application have the same specification for the mean of *y*_*ij*_ as those previously formulated in Loquiha *et al* [33]. Specifically, we consider the following initial model for the mean *μ*_*ij*_:

$$\begin{array}{c} \begin{array}{cc} \text{log}(\nu_{ij}) & =\beta_{0}+\beta_{1}NORTH_{ij}+\beta_{2}CENTER_{ij}+\beta_{3}PH_{ij}+\beta_{4}HC1_{ij}+\beta_{5}HC2+\beta_{6}RH_{ij}\\ & +\beta_{7}LOC_{ij}+\beta_{8}WAIT_{ij}+\beta_{9}MED_{ij}+\beta_{10}EMOC_{ij}+\beta_{11}MAL_{ij}+\beta_{12}HIV_{ij}\\ & +\beta_{13}REFOUT_{ij}+\beta_{14}REFIN_{ij}\\ & +\beta_{15}NORTH\times LOC_{ij}+\beta_{16}CENTER\times LOC_{ij}+\text{log}\left({\text{N}}_{ij}\right)\\ \text{logit}(\pi_{ij}) & =\alpha_{0}+\alpha_{1}NORTH_{ij}+\alpha_{2}CENTER_{ij}+\alpha_{3}LOC_{ij}+\alpha_{4}MAL_{ij} \end{array} \end{array}$$ (7)

where NORTH and CENTER are two dummy variables for the regions (South = reference, Center, North); LOC refers to location of HF (district capital = reference, outside capital); PH, HC1, HC2 and RH are 4 dummy constructs for type of health facility (central hospital = reference, PH = provincial hospital, HC1 = health center I, HC2 = health centers II/III/h.post and RH = rural hospital), WAIT refers to waiting house (not available = reference, available), MED is ratio of medical doctors, EMOC refers to emergency obstetric care (none = reference, partial/full time), MAL refers to proportion of malaria cases, HIV to proportion of HIV cases, REFOUT to referral to other HFs and REFIN to referral from other HFs. This model construction was a result of a likelihood-based backward regression procedure with significance level for the removal set at 0.20.

Table 2 shows the DIC and Brier score for the best fitting models. Other models were also estimated, but since their fits were inferior, their results are not reported here. The negative binomial family of models seemed to outperform its Poisson equivalent, except when the hierarchical structure of the data is taken into account. The simple Poisson regression showed the worst fit of all models considered with a DIC = 2015.2 versus DIC = 1055.9 of the simple negative binomial regression, once again highlighting the need for properly accounting for overdispersion in the model. We observed a much greater reduction on the DIC or Brier score when the ZIP models incorporate random effects than when the ZINB models do. The HZIP (correlated) ranked as the best model when spatial effects were ignored, with a DIC of 927.3 and Brier score of -0.3415, followed closely by the HZIP (independence) with a Brier score equal to -0.3441, not surprisingly so since the (non-spatial) between-component correlation was estimated at 0.44 (95% credible interval(CI): [-0.28; 0.86]) which was statistically not different from zero.

**Table 2:** Model fit summary for best zero inflated models.

Model	Deviance	Effective par.	DIC	Brier score
*HZIP (correlated)*	841.8	85.4	927.3	-0.3415
*SpHZIP (correlated)* ^1^	840.4	87.4	927.8	-0.3413
*SpHZIP (correlated)* ^2^	840.4	85.3	925.6	-0.3414
*SpHZIP (correlated—independence)* ^3^	837.9	91.8	929.6	-0.3397
*SpHZIP (correlated—correlated)* ^3^	833.3	91.7	924.9	-0.3397

“H” stands for hierarchical or random effects model and “Sp” for spatial.

^1^: with intrinsic CAR assumption

^2^: with proper CAR assumption

^3^: with intrinsic bivariate CAR assumption

When spatial effects are considered using an intrinsic CAR prior, we observed a similar pattern as before: SpHZIP models improved the fit of a simple spatial Poisson regression and they offered better fits than the SpHZINB, with both DIC and Brier scores. Again, the SpHZIP (correlated) is the best model with a score of -0.3413, which is not that different to when the spatial structure was ignored. In fact, the DIC value slightly increased, from 927.3 when spatial effects were ignored to 927.8 when spatial effects were included. A preliminary conclusion here is that spatial heterogeneity is not significant or is already taken into account with the incorporation of non-spatial random effects. Also, a global Moran’s I test for which the statistic was equal to 0.077 with p-value = 0.0720, was indicative of no positive spatial autocorrelation of mortality rates across areas in Mozambique. Looking at the variance components estimates for the SpHZIP (correlated) in Table 3, the variance of *θ* (random intercept on number of maternal deaths) estimated as 1.24 is roughly 2 times the variance of *υ*_2_ (spatial random effect on number of maternal deaths) at 0.79, indicative once more for the dominance of non-spatial heterogeneity compared to the spatial one. Also, for this model there was no sufficient evidence for between-component correlation ($\hat{\rho }=0.44$, 95% CI: [-0.37; 0.87]). This is also the case when proper CAR priors are considered, with the best model SpHZIP (independence) having a score of -0.3413 followed closely by the SpHZIP (correlated) with a score of -0.3414, and *ρ* statistically not different from zero (0.44 and 95% CI: [-0.32; 8.87]). As pointed out previously, spatial patterns or clusters on the NMNH data cannot be completely identified by only a spatial random effect on the counts component of a zero-inflated model. This is shown by the improvement obtained in model fit when bivariate CAR priors are considered. Although the SpHZIP(correlated—correlated) had the lowest DIC value (924.9), we obtained the exact same Brier score as for the SpHZIP (correlated—independence) of -0.3397, implying no spatial dependence between model components. The estimate of $\sigma_{\upsilon_{12}}$ was -0.54 (95% CI: [-1.88; 0.11]) which shows a negative between-component association, i.e., areas with high likelihood of maternal deaths tend to show a reduced number of facility-based maternal deaths, but with no sufficient evidence that this is indeed different from zero. However, an interesting note about this model is the considerable variation of spatial random effects introduced in the zero component relative to its equivalent in the counts component. From these results, a much simpler model was constructed through a model building process starting from the SpHZIP(correlated—correlated) model. We also removed non-significant fixed effects and correlation that had been encountered in the previous models and end up with the more parsimonious SpHZIP (independence—independence) model, which assumes that a multivariate set of independent random intercepts and spatial effects in each model component account for non-spatial and spatial heterogeneity, respectively. The 95% credible intervals for the variances of spatial random effects were wider than their non-spatial equivalents, and not bounded away from zero, which may lead to questioning their statistical significance. The same can be said regarding the relevance of *ϑ* given the wider 95% CI: [0.01; 1.42] relative to the posterior estimate of the variance of 0.26.

**Table 3:** Posterior estimates (95% credible interval) for variance components of the best 4 models with and without spatial effects.

Effect	HZIP (corr)	SpHZIP(corr)^1^	SpHZIP(corr)^2^	SpHZIP(corr-corr)^3^	SpHZIP(ind-ind)^4^
$\sigma_{\theta }^{2}$	1.60(1.02, 2.43)	1.24(0.42, 2.15)	1.20(0.71, 1.86)	1.34(0.76, 2.29)	1.09(0.34, 2.05)
*ρ*	0.44(-0.28, 0.86)	0.44(-0.37, 0.87)	0.41(-0.32, 0.87)	0.55(-0.16, 0.91)	—
$\sigma_{\vartheta }^{2}$	0.77(0.15, 2.40)	0.86(0.16, 2.50)	0.74(0.15, 2.19)	0.95(0.15, 3.12)	0.26(0.01, 1.42)
$\sigma_{\upsilon_{1}}^{2}$	—	—	—	1.23(0.11, 4.56)	0.84(0.01, 3.95)
$\sigma_{\upsilon_{12}}$	—	—	—	-0.54(-1.88, 0.11)	—
$\sigma_{\upsilon_{2}}^{2}$	—	0.79(0.04, 3.07)	0.31(0.00, 2.43)	0.53(0.04, 1.64)	2.05(0.08, 6.07)
*ψ*	—	—	-0.10(-0.74, 0.97)	—	—

^1^: with intrinsic CAR assumption

^2^: with proper CAR assumption

^3^: with intrinsic bivariate CAR assumption (correlated-correlated)

^4^: with intrinsic bivariate CAR assumption (independence—independence)

Results for the fixed effects of the SpHZIP (independence—independence) model are presented in Table 4. Posterior means for the binomial component of the model, showed that only HF location is strongly associated with the propensity for facility-based maternal deaths. The odds for reporting no maternal deaths was roughly 21 times (exp(3.04) = 20.91, 95%*CI*:[7.61;75.94]) higher when the HF was located outside the district capital (i.e., in rural areas) compared to inside the district capital. On the other hand, the expected number of maternal deaths in central hospitals was higher than in any other health facility type, being as much as 93% higher (exp(−2.78) = 0.06, 95%*CI*:[0.03;0.12]) when compared to health center II.

**Table 4:** Posterior estimates (95% credible interval) for fixed effects in the SpHZIP(independence—independence) model.

Effect (reference category)	SpHZIP(ind-ind)
Model for logit(*π*_*ij*_)	
*Intercept*	-2.11(-3.29, -1.12)
*Location (district capital)*	
Outside capital	3.04(2.03, 4.33)
Model for log(*ν*_*ij*_)	
*Intercept*	-3.17(-3.70, -2.56)
*Facility type (Central hospital)*	
Provincial hospital	-0.32(-0.52, -0.12)
Health center I	-1.87(-2.24, -1.54)
Health center II/III/H.Post	-2.78(-3.39, -2.15)
Rural hospital	-1.33(-1.88, -0.79)
*Waiting house (Not available)*	
Available	-0.79(-1.20, -0.39)
*Ratio of medical doctors*	1.12(0.17, 2.13)
*Emergency obstetric care (None)*	
Full time	-0.76(-1.24,-0.29)
*Proportion of referrals to*	-2.57(-3.13, -2.03)
*Proportion of referrals from*	-3.62(-7.01, -0.87)

DIC = 951.1

Brier score = -0.3407

Also, the availability of a waiting house reduced the expected number of maternal deaths by about 55% (exp(−0.79) = 0.45, 95%*CI*:[0.30;0.68]), similar to availability of full time emergency obstetric care (53%, exp(−0.76) = 0.47, 95%*CI*:[0.29;0.75]). Interestingly, the more medical doctors a facility has, the higher the average number of maternal deaths (as high as 3 times, exp(1.12) = 3.06, 95%*CI*:[1.19;8.42]). This is to be expected, since a higher proportion of medical doctors are located in central hospitals, usually in major cities.

**Fig 4:**
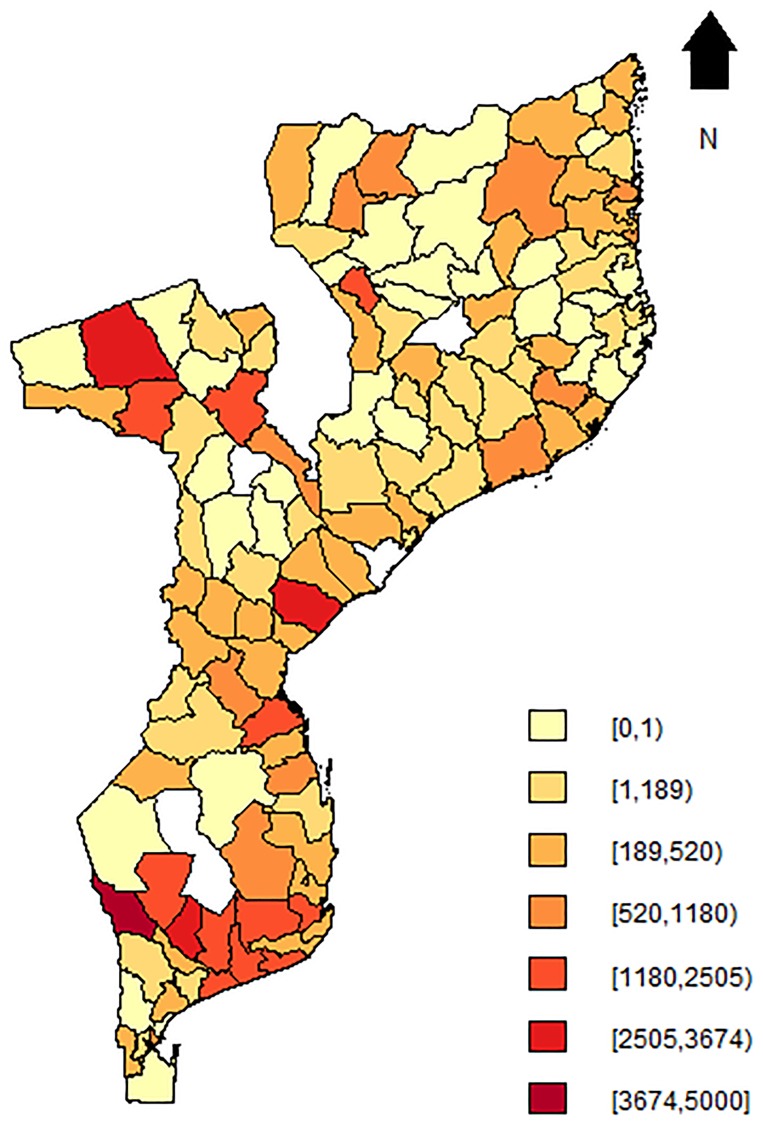
Map of posterior means of maternal mortality rate based on the SpHZIP (independence-independence) model. Blank spots indicate districts for which data was not available.

**Fig 5:**
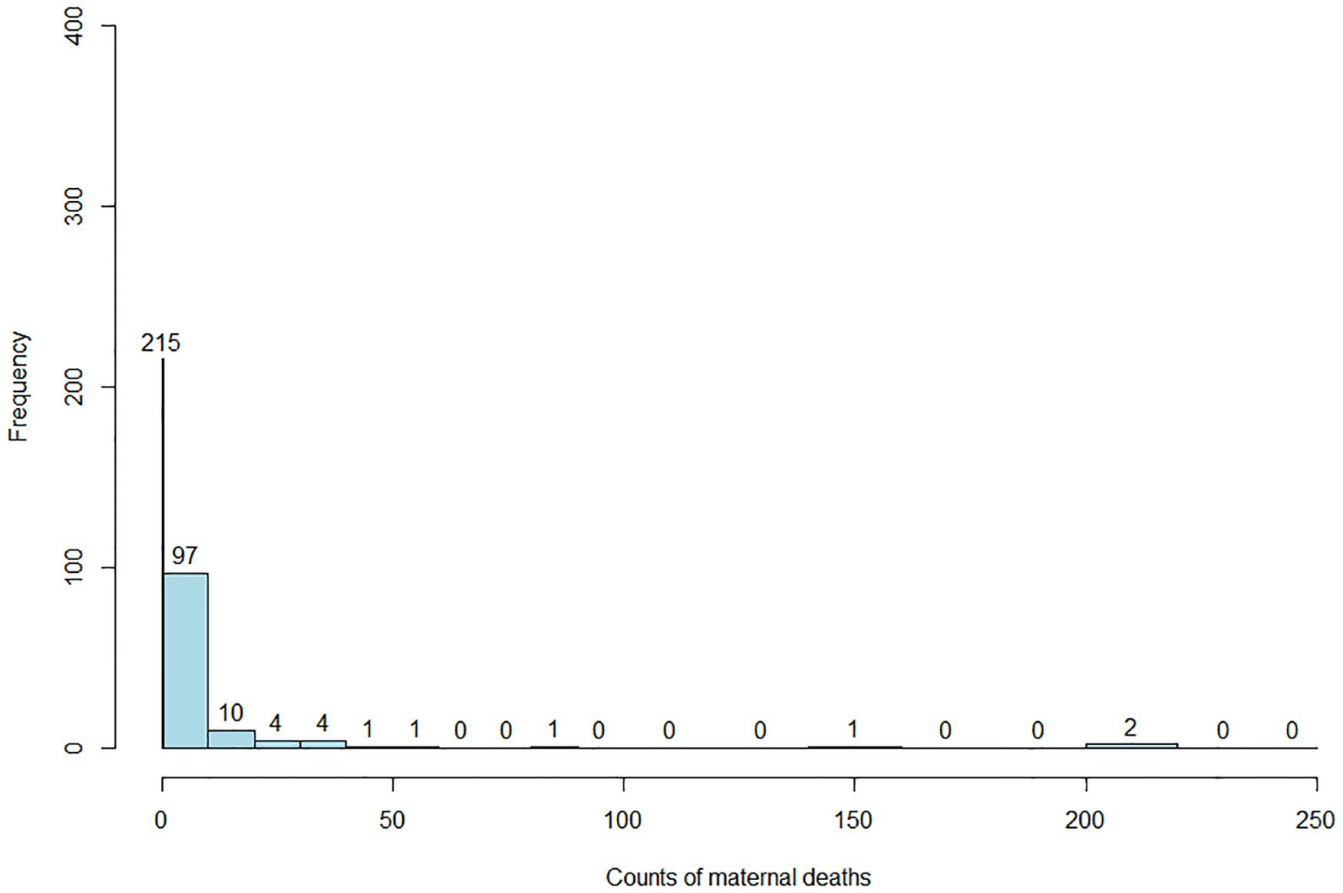
Histogram of posterior predictive counts of maternal deaths based on the SpHZIP (independence—independence) model.

Fig 4 presents the map for the predicted maternal mortality rate (${\hat{\lambda }}_{i}$) from the SpHZIP (independence—independence) model, calculated by aggregating the predicted counts and dividing it by the total number of obstetric admissions from each district (× 100,000). The maternal mortality rate based on posterior predictions of the model showed a very similar spatial pattern as observed with the crude mortality rate (Fig 3), though slightly smoothed as a result of borrowing information from neighboring districts. Again, districts in the South showed the highest mortality rate, followed by districts in the Center and lastly the North. The district of Massingir in the Gaza province (South) continues to show the highest facility-based maternal mortality rate of 3843.5 (per 100,000 obstetric admissions), about 19.1% lower then the observed rate. In Fig 5, we show the histogram of predicted counts of facility-based maternal deaths. Overall, the model fits the data quite well, with the predicted counts being close to the observed counts as shown in Fig 2.

**Fig 6:**
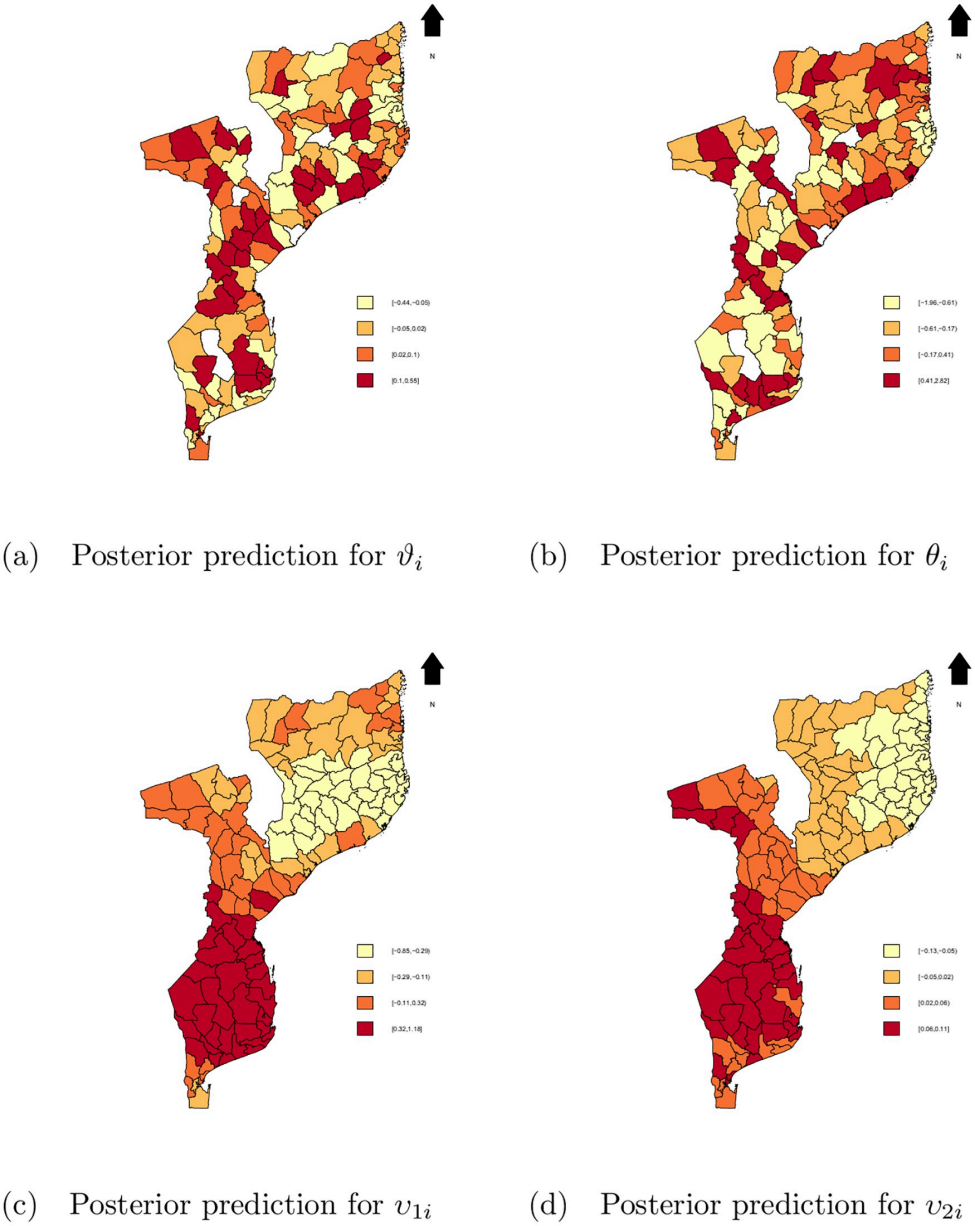
Maps of posterior mean. a: for *ϑ*_*i*_, b: for *θ*_*i*_, c: for *υ*_1*i*_ and d: for *υ*_2*i*_ based on the SpHZIP (independence—independence) model. Blank spots indicate districts for which data was not available.

Fig 6 shows the posterior predictive distributions of non-spatial and spatial random effects. There was more variation, geographically, in the non-spatial random effects, presented on Fig 6a and 6b, contrary to the spatial effects on Fig 6c and 6d. The geographical distribution of non-spatial random effects is a mirror of the distribution for the observed and predicted mortality rate, where roughly the same set of districts showed increased propensity for institutional maternal deaths or increased expected counts of maternal deaths as before. The distribution of spatial random effects, however, shows huge clusters of effects structured by regions: South region with highest effects, reducing as we move to the North. Recall that dark colors indicate districts with elevated propensity for institutional maternal death or increased expected counts of maternal deaths compared to an “average” or “typical” district, i.e, when random effects = 0, given the same set of covariates.

## Discussion

In this paper, we extended the ZIP and ZINB models used in [33] to address the need for sharing information between neighboring areas when modeling facility-based maternal mortality rate in Mozambique. Results showed that using the bivariate intrinsic CAR specification for spatial random effects into zero-inflated models that already account for correlated count data slightly improved the fit, and that this is more pronounced when using the Poisson distribution, a surprising result based on our findings from [33] where the Negative binomial distribution outperformed the Poisson distribution for any considered extension. Although the best model formulation allowed an estimation of both spatial and non-spatial within and between-components correlation in a zero-inflated setting, more complex models need not always be preferred, specially if similar fits can be accomplished with relatively simpler models. This is the case in this application as was also in Silesh *et al*. [17] and Neyens *et al*. [30].

An independence structure was imposed for the multivariate distribution of spatial and non-spatial random effects but it is difficult to imagine a situation where more complex structures were necessary, as there may not be enough information in the data to attribute to various sources of variability. For instance, we found that there was no sufficient variability in the data to support spatial and non-spatial between-component correlations. Also, with a high proportion of structural zeros in the NMNH data (zeros from health center type II/III and health posts) the question on whether to add random effects to the binomial component of the model is no longer trivial and other statistical tools need to be considered in the verification of adequacy of random effects [31, 43]. What’s more, the random-intercepts model specification implies an equal within-district correlation assumption, meaning that the correlation of counts of larger or smaller health facilities is the same within districts. This might be problematic if smaller sites consistently reported 0 maternal deaths. The results showed no evidences that the probability for reporting zero maternal deaths was related to the type of health facility, but rather to its location (outside district capital vs inside district capital). It then seemed reasonable to ignore the type of health facility in any correlation structure formulation and assume equal correlation within districts conditional on either the health facility reports 0 maternal deaths or 1 or more maternal deaths.

Our application assumed the data to be missing completely at random (MCAR), and so a complete case analysis was performed. Although no test was performed to check the MCAR assumption it seemed reasonable to believe that it holds since this specific data was aggregated and derived from administrative records which in the case of Mozambique may lack for proper management. Methodologies to deal with non-ignorable missingness in non-spatial zero-inflated models are provided in Hasan *et al*. [44] and Maruotti [45]. However, a careful handling of the missing data was a task beyond the scope of this paper.

Maps were used to highlight areas with increased and reduced mortality rate and, in general, such areas were located in the South and North of Mozambique, respectively. Because the non-spatial variation, related to the unstructured random effects *θ*_*i*_ and *ϑ*_*i*_ was larger relative to the spatial variation (related to *υ*_1*i*_ and *υ*_2*i*_), as observed in the estimated covariance matrix for SpHZIP (independence-independence) model, there was not much smoothing in the maps of the maternal mortality rates, despite the elevated spatial effect presented in the South and central regions of Mozambique. Regional inequalities play an important role in explaining the inefficacies found in the health system in Mozambique. Historically, the South region of Mozambique is more developed than the other 2 regions, with many more urban areas and health facilities. Our intuition is that what these results show is not the need to increase or strengthen the health system in the South region, but the historical inequality of health care use between the regions in Mozambique. This is supported by the results of the SpHZIP(correlated—correlated) model which showed that the expected counts of maternal deaths for health facilities in the North region and located outside the district capital is 93% lower compared to health facilities in the South located inside the district capital. However, the expected counts for facilities in the central region and outside the district capital is 27% higher compared to facilities in the South, although overall, counts in the central region were expected to be approximately 12% lower than in the South.

## Supporting information

S1 File The NMNH survey data file. (CSV)

S1 Appendix WinBUGS codes for SpHZIP (correlated—correlated) model. (PDF)
