# Supplementary material for: Mapping maternal mortality rate via spatial zero-inflated models for count data: A case study of facility-based maternal deaths from Mozambique
Source: PLoS One. 2018 Nov 9;13(11):e0202186. doi: 10.1371/journal.pone.0202186 (PMC6226154; doi:10.1371/journal.pone.0202186)
Supplement: S1 Appendix — (PDF) [file pone.0202186.s002.pdf]

Mapping maternal mortality rate via spatial zero-inflated models for count data: a case study of facility-based maternal deaths from Mozambique

S1 Appendix. WinBUGS codes for SpHZIP (correlated - correlated) model

```
model{
C<-0
for (i in 1:N){
zeros[i]<- 0
zeros[i]~dpois(zero.mean[i])
zero.mean[i]<- -l[i]+C

#expression of log-likelihood for observation i
l[i]<- log(pi0[i]*equals(y[i],0) + (1-pi0[i])*fd[i])

#density function for Poisson distribution
fd[i]<- exp(-lambda[i] + y[i]*log(lambda[i]) - loggam(y[i]+1))

#expressions for linear predictors
logit(pi0[i])<-alpha[1]+alpha[2]*x1[i]+alpha[3]*x2[i]+alpha[4]*x7[i]
+alpha[5]*x11[i]+v[id[i],1]+sp[1,id[i]]
log(lambda[i]) <- beta[1]+beta[2]*x1[i]+beta[3]*x2[i]
+beta[4]*x3[i]+beta[5]*x4[i]+beta[6]*x5[i]+beta[7]*x6[i]+beta[8]*x7[i]
+beta[9]*x8[i]+beta[10]*x9[i]+beta[11]*x10[i]+beta[12]*x11[i]
+beta[13]*x12[i]+beta[14]*x13[i]
+beta[15]*x14[i]+beta[16]*x1[i]*x7[i]+beta[17]*x2[i]*x7[i]+log(x15[i])
+v[id[i],2]+sp[2,id[i]]
mu[i]<-(1-pi0[i])*lambda[i]
}

#model parameter priors
for (k in 1:5){
alpha[k]~ dnorm(0,tau.alpha)
}
for (k in 1:17){
beta[k]~ dnorm(0,tau.beta)
}
#hyper-priors
tau.alpha~dgamma(1.0E-5,1.0E-5)
tau.beta~dgamma(1.0E-5,1.0E-5)

#non-spatial priors
for(j in 1:128){
v[j, 1:2]~ dmnorm(zero[], prec.Sigma[,])
}

# spatial priors
```

```
sp[1:2, 1:128] ~ mv.car(adj[], weights[], num[], omega.sp[,])

# prior on precision matrix for spatial effects
omega.sp[1:2,1:2] ~dwish(Q[,],2)
for (h in 1:2) {for (f in 1:2) {Q[h,f] <- equals(h,f)*0.1}}
# covariance matrix
Sig[1:2,1:2] <- inverse(omega.sp[,])
rho.sp <- Sig[1,2]/sqrt(Sig[1,1]*Sig[2,2])

#covariance matrix for the non-spatial random effects
Omega[1,1] <- 1
Omega[2,2] <- 1
Omega[1,2] <- 0
Omega[2,1] <- 0
prec.Sigma[1:2, 1:2] ~ dwish(Omega[,], 2)
Sigma[1:2,1:2] <- inverse(prec.Sigma[,])
zero[1]<-0
zero[2]<-0
rho<-Sigma[1,2]/sqrt(Sigma[1,1]*Sigma[2,2])

#Briers score
for (j in 1:1){
for (z in 1: N){
# if y>0 branch
ppo_branch[z, 1]<-(1-pi0[z])*exp(-lambda[z] + y[z]*log(lambda[z])
- loggam(y[z]+1))
# else branch
ppo_branch[z, 2]<-pi0[z]+(1-pi0[z])*exp(-lambda[z] + y[z]*log(lambda[z])
- loggam(y[z]+1))
if_branch[z] <- 1 + step(-(y[z] - 0.5))
# 1 for "then" branch, 2 for "else" branch
ppo[z]<- ppo_branch[z, if_branch[z]]
Bsc[j,z]<-pow(ppo[z]-equals(y[z],y[z]),2)
}
B.score[j]<--sum(Bsc[j,1:N])/N}
}
```
